# The Utilization of Physician Cell Phone Numbers by Patients in an Orthopaedic Surgery Practice

**DOI:** 10.7759/cureus.7712

**Published:** 2020-04-17

**Authors:** Ryan G Rogero, Meghan Bishop, Brandon J Erickson, Daniel Seigerman, Daniel Smith, Samir C Sodha, Howard Yeon, Justin Tsai

**Affiliations:** 1 Orthopaedic Surgery, Rothman Orthopaedic Institute, Philadelphia, USA; 2 Orthopaedic Surgery, Lewis Katz School of Medicine at Temple University, Philadelphia, USA; 3 Orthopaedic Surgery, Rothman Orthopaedic Institute, New York, USA

**Keywords:** cellular phone, communication, technology, orthopaedic surgery

## Abstract

Introduction

Orthopaedic surgeons choose to manage communication with their patients outside of official visits and interactions in a variety of ways, with some choosing to provide their personal cell phone number in order to provide patients with direct accessibility. The objective of this prospective study is to explore to what extent patients utilize the cell phone numbers of orthopaedic surgeons in the immediate period after it is provided to them.

Methods

Seven fellowship-trained orthopaedic surgeons from five different subspecialties in a single private, multi-site group each provided his/her personal cell phone number to 30 consecutive patients. The surgeon’s phone number was written down on a business card, and the surgeons themselves provided the card to the patient. Phone calls and voice mail messages received in the 30 days following the patient receiving the phone number were recorded, and the reasons for these calls were categorized as being “appropriate” (e.g. acute postoperative issues, unclear instructions) or “inappropriate” (e.g. administrative issues, medication refills, advanced imaging-related inquires).

Results

Two-hundred seven patients with an average age of 51.5 years were provided cell phone numbers. During the 30 days following administration of cell phone numbers to each patient, 21 patients (10.1%) made calls to their surgeons, for an average of 0.15 calls per patient. Six patients (2.9%) called their surgeons more than once. Seventeen calls (54.8%) were deemed appropriate, while 14 calls (45.2%) were inappropriate. Logistic regression analysis did not reveal patient age, sex, type of visit, or surgeon subspecialty to be independently associated with calling.

Conclusion

Our study has demonstrated a low rate of patient utilization of surgeon cell phone number when provided to them. If surgeons choose to provide their cell phone number to patients, we recommend specifying appropriate reasons to call in order to maximize the effectiveness of this communication method.

## Introduction

Although the use of the cell phones and smartphones in healthcare has increased over the past decade, most studies and reports have focused on physician utilization [1]. Examples include mobile applications as references for diagnoses, surgical approaches and techniques, physician networking, medication dosage guidelines and others [2-5]. However, there has been comparatively little research done on how the use of cell phone/smartphone by the patient has influenced patients’ communication with their physician [6]. The importance of effective communication between the patient and physician has certainly been well-documented in the literature [6-8]. This is perhaps even more so in patients undergoing surgical procedures, who often find themselves in uncharted territory pre- and postoperatively [9].

Anecdotally, there are a wide variety of ways in which physicians choose to manage communications with their patients outside of official visits and interactions. Some defer to ancillary staff (e.g. surgery schedulers, medical assistants) or mid-level providers (e.g. registered nurses, physicians assistants) until the situation warrants a call back directly from the physician. Less commonly, some choose to provide their cell phone number to patients to provide them with direct access should the need arise [6,10]. Despite the possibility of what might be perceived as inappropriate use of this information, it is our experience that inappropriate utilization of physician cell phones by patients rarely occurs [10,11].

The purpose of this prospective study is to explore to what extent patients utilize physician cell phone numbers when provided to them. The authors hypothesized that less than 25% of patients would utilize this resource and that the majority of these calls would be for appropriate reasons.

## Materials and methods

Institutional review board approval was obtained prior to the initiation of this study. In this prospective study, seven fellowship-trained orthopaedic surgeons from five different subspecialties (adult reconstruction, foot and ankle, hand and wrist, spine, sports) in a single private, multi-site orthopaedic surgery group located in a major metropolitan area each provided their personal cell phone number to approximately 30 consecutive patients during a one-month period. Consecutive patients were used in order to eliminate possible surgeon bias as to which patients were more or less likely to call inappropriately. The surgeon’s phone number was written down on a business card, and the surgeons themselves provided the card to the patient, informing the patient that they “can and should call with any questions, concerns, or issues”. Unless prompted, no distinction was made as to what situations were appropriate for calling the physician as opposed to calling ancillary staff or mid-level providers. Patients were instructed to call rather than send an SMS text message. No other change in the typical office protocol was implemented by participating surgeons. In accordance to practice protocol, following every phone call, the reason for the call was then documented in the electronic medical record. Phone calls and voice mail messages received by surgeons on their personal cell phones in the 30 days following the patient receiving the phone number were immediately recorded following each call by each surgeon into the database, and the reasons for these calls were categorized as being “appropriate” or “inappropriate” using predetermined criteria (Table 1).

**Table 1:** Guidelines as to what constituted an appropriate or inappropriate call during the study period

APPROPRIATE
Acute postoperative issues – increasing pain, drainage, etc.
Unclear postoperative instructions
NOT APPROPRIATE
Administrative issues – inquiring about the time of appointment, issues with a bill, treatment costs, etc.
Inquiring about instructions clearly delineated on discharge instructions
Discuss advanced imaging orders/results
Medication refills

Patient charts were retrospectively reviewed to record patient age, sex, and type of visit (new patient, established patient, postoperative, or imaging result visit) when provided the cell phone number.

Descriptive statistics (mean, standard deviation, ranges) were performed on patient demographics. Student’s t-tests and chi-square analysis were performed to determine whether any patient variables were different between callers and non-callers. Logistic regression analysis was performed to determine if any patient variable or surgeon subspecialty was independently associated with calling the surgeon cell phone number. Statistical significance was set at P≤0.05.

## Results

Two-hundred seven patients (59.4% female) with an average age of 51.5 ± 19.5 years were provided orthopaedic surgeon cell phone numbers (Table 2).

**Table 2:** Patient demographic variables and comparison of patient and surgeon factors on calling ^a^Values reported as mean ± standard deviation or N (%)

Variable		Overall (N=207)^a^	Called (N=21)	No Call (N=186)	P
Age (years)		51.5 ± 19.5	53.0 ± 19.4	51.4 ± 19.5	0.7174
Sex	Female	123 (59)	12 (57)	111 (60)	0.8191
	Male	84 (42)	9 (43)	75 (40)	
Visit Type	New patient	101 (49)	11 (52)	90 (48)	0.3118
	Existing patient	54 (26)	5 (24)	49 (26)	
	Postoperative	39 (19)	2 (10)	37 (20)	
	Test/imaging result	13 (6)	3 (14)	10 (5)	
Subspecialty	Adult reconstruction	30 (14)	2 (10)	28 (15)	0.3119
	Foot and ankle	25 (12)	1 (4)	24 (13)	
	Hand	63 (30)	5 (24)	58 (31)	
	Spine	30 (14)	3 (14)	27 (15)	
	Sports	59 (29)	10 (48)	49 (26)	

During the 30 days following receipt of cell phone numbers, 21 patients (10.1%) made calls to their surgeon, for an average of 0.15 calls per patient. Six patients (2.9%) called their surgeons more than once. Based on the categorization criteria agreed upon by the surgeons in the study, 17 calls (54.8%) were deemed appropriate, while 14 calls (45.2%) were deemed inappropriate (Table 3).

**Table 3:** Summary of inappropriate reasons for calling

Reason for Call	No. of Calls
Return to work question/request	3
Advanced imaging scheduling/authorization	2
Non-urgent postoperative period question	2
Advanced imaging question	1
Called by mistake	1
Confirming imaging results received	1
Cost of treatment question	1
Provided further history of the current condition	1
Re-explanation of previously answered questions	1
Surgical plan question	1
Total	14

The inappropriate reasons for calling occurring more than once included calls regarding return to work (n=3/14, 21.4%), advanced imaging scheduling/authorization (n=2/14, 14.3%), and non-urgent postoperative period questions (n=2/14, n=14.3%). Providing a further history of current condition, re-explanation of a previously answered question, confirmation of received imaging results, calling by mistake, and questions regarding advanced imaging, cost of treatment, and surgical plan all each occurred once (n=1/14, 7.1%) during the study period.

Student’s t-tests (age) and chi-square analysis (sex, type of visit, surgeon subspecialty) did not reveal any significant difference between callers and non-callers (Table 2). Sports had the highest proportion of patients calling (16.9%), whereas foot and ankle had the lowest (4.0%), though surgeon subspecialty did not differ significantly (P=0.3119). Logistic regression analysis did not reveal patient age (P=0.7147), sex (P=0.8230), type of visit (P=0.8745), or surgeon subspecialty (P=0.1960) to be associated with calling the surgeon.

## Discussion

In the present study, we have attempted to elucidate the rate of utilization of the personal cell phone numbers of orthopaedic surgeons when provided to patients. During our study period, 21 patients (10.1%) made 31 calls, which confirmed our hypothesis of a less than 25% utilization rate. The majority of these calls (54.8%) were deemed appropriate, which also aligned with our hypothesis.

The importance of physician-patient communication is well-recognized, and its role continues to evolve as the healthcare system transitions to an outcome-based model with heavy reliance on patient satisfaction [12]. Physician accessibility constitutes an essential part of this relationship, and availability beyond in-person visits can be argued to be as equally as important in patient care [10]. Physicians who are more accessible have also been shown to have more satisfied patients who are more likely to be compliant with instructions as well as less litigious [10,13]. With the drastic rise in smartphone and cell phone ownership in recent years, this technology has an enormous potential for further extending the methods of communication and physician accessibility that currently constitutes the physician-patient relationship [12]. To the authors’ knowledge, this prospective study involves the largest series of patients being given the cell phone number of their orthopaedic surgeons and the first including patients from multiple perioperative visit types and seeing physicians from different orthopaedic surgery subspecialties.

Better methods of communication with orthopaedic surgeons have been consistently reported by patients. Chin et al. surveyed 210 patients on the impact of receiving their doctor’s cell phone number and found that 85% of patients would consider using the surgeon’s number [6]. The authors concluded this demonstrated a large desire for patients to have direct access to their surgeon. A recent study surveying expectations of communication during the perioperative period of joint-sparing elective knee surgery reported that 63% of patients felt it “important” or “very important” that the operating surgeon was available by phone after surgery compared to only 32% feeling “important” or “very important” of surgeon email availability [14]. Further, when surveying 310 patients in an urban academic orthopaedic practice regarding preferences for an orthopaedic smartphone app, Datillo et al. found that the ability to communicate with the physician team was one of the most desired features, just trailing appointment reminders and the access to test or procedure results [12].

The utilization of telephone and text message patient follow-up and smartphone applications within the orthopaedic field is common [5,9,15-19]. However, despite the immense presence of cell phones as a primary method of communication within our daily lives, a limited investigation into their potential role in orthopaedic physician-patient communication has previously been performed [6]. Chin et al. found that when 32 patients were provided the phone numbers of the surgical scheduler, the surgeon’s secretary, and the surgeon, only 12 of 65 calls (18.5%) received during their study period were made to the surgeon’s personal number [6]. Hällfors et al. examined the use of a consultation phone service for postoperative total joint arthroplasty patients, concluding that the service could effectively address patient concerns and cut down the number of emergency department visits and subsequent healthcare costs [20]. Day et al. reported an increased level of patient satisfaction and a better understanding of health responsibilities with the use of a perioperative automated mobile phone messaging consisting of reminders, activity, and pain control [9].

In the current study, we have demonstrated a 10.1% rate of surgeon cell phone utilization, which aligned with our hypothesis of a low utilization rate. Further, the majority of these calls (54.8%) were deemed appropriate, though a substantial portion of the calls was for inappropriate reasons. Additionally, none of the 65 calls made during their study period were made during evening hours [6]. In order to further tailor the utilization of this resource, we suggest explaining to patients verbally or in writing about what constitutes an appropriate call to the surgeon’s personal phone number, such as acute postoperative issues and unclear postoperative instructions. Providers can also instruct patients to leave a voicemail message that specifies the reason for their call so that physicians may directly respond to the appropriate matter, while designating an ancillary staff member to respond to all “inappropriate” calling reasons. Additionally, we recommend explaining to patients that if they are unable to reach their surgeon for any potential urgent or emergency situations, they should contact the office call center or emergency services immediately. Of note, no patients in this study utilized physician phone numbers for questions regarding emergency type situations.

We did not find any factors, including age, sex, visit type, or orthopaedic subspecialty, to be associated with increased rates of calling the surgeon’s personal number. In regard to the type of visit, patients being given the cell phone number at new patient visits (52%) constituted the majority of calls made, which may have been expected, as patients may have considered being given the surgeon’s number a regular practice at the institution with limitless restrictions. The sports subspecialty had the largest proportion of patients calling (16.9%), while foot and ankle had the lowest (4.0%); however, subspecialty was not shown to differ based on the chi-squared analysis. Though no evidence in the literature was found comparing call rates across subspecialties, Hadeed et al. found that lower extremity procedures constituted the highest procedures for patient-initiated telephone calls during the first 14 days after discharge following orthopaedic trauma procedures [21].

The major strengths of this study include a large number of patients to whom phone numbers were provided as well as the involvement of surgeons from several different orthopaedic subspecialties. Patients presenting for a variety of visit types were also enrolled, thereby extending the patient cohort between the more commonly exclusively investigated postoperative patient population. Detailed documentation of the reasons for calling was also performed by the surgeons involved in the study, thereby giving other surgeons a better understanding of some of the reasons patients may call for if they should choose to give out their personal phone number.

Several limitations do exist with this study. First, we only recorded phone calls for a time period of 30 days following administration of the surgeon’s cell phone number. A longer time would have potentially allowed for further assessment of this communication resource, but we felt patients were more likely to call within this given period and that this utilization rate would be similar if not decreased if the follow-up period was extended in duration. Previous studies have used similar follow-up periods [6,19,21]. There is also a likelihood that not all calls to the surgeon’s personal number were recorded, as patients may not have left a voicemail or callback number if unable to directly reach the surgeon. We also did not record the number of phone calls made by patients to ancillary staff providers, which would have allowed a quantitative comparison of calls made to each phone number. Additionally, our patient numbers may have been too low to detect statistical differences between patient age, sex, type of visit, or surgeon subspecialty, though, to our knowledge, 207 patients are the largest cohort examining this topic. Finally, we were unable to assess patients’ experiences with being given their surgeons’ personal phone number, but future studies should attempt to carry this out in order to evaluate this method of communication on patient satisfaction and perception of care.

## Conclusions

Despite the aforementioned limitations, we have demonstrated that providing physician cell phone numbers can be an effective method of communication and a way to efficiently fulfill the desire of orthopaedic patients to have better communication with their physicians. We further suggest specifying to patients what should be considered an appropriate call to surgeons in order to maximize the potential of this resource.
